# Awareness and use of a community health worker program among racial/ethnic minority adults in Philadelphia: Results from a cross‐sectional community health survey

**DOI:** 10.1002/ajcp.70092

**Published:** 2026-07-28

**Authors:** Stephen Bonett, Carmen Alvarez, José Bauermeister, Ashley Clemmons, Hyunmin Yu, Andy S. L. Tan, Terri H. Lipman, Karen Glanz, Antonia M. Villarruel

**Affiliations:** ^1^ University of Pennsylvania School of Nursing Philadelphia Pennsylvania USA; ^2^ City of Philadelphia Office of Community Empowerment and Opportunity Philadelphia Pennsylvania USA; ^3^ University of Pennsylvania Annenberg School for Communication Philadelphia Pennsylvania USA; ^4^ University of Pennsylvania Perelman School of Medicine Philadelphia Pennsylvania USA

**Keywords:** community health workers, health equity, healthcare access, program evaluation

## Abstract

Community health workers (CHW) programs are effective at reducing hospitalizations, improving mental health, and supporting health‐promoting behaviors. Research suggests awareness and use of CHW services remain uneven across communities nationally. This study describes awareness, use and satisfaction with a city‐wide CHW program among racial/ethnic minority individuals in Philadelphia. Data come from a web‐based health survey conducted February–March 2024. Participants reported sociodemographic characteristics, health conditions, barriers to social resources, and awareness and use of the city's CHW program. Among 522 participants, the majority were Black (55.4%), women (70.1%), with mean age 39 years. Less than half (44.6%) were aware of the CHW program; of those, 30.5% (13.6% of whole sample) had used it. Among users, 80.3% reported being satisfied with services. In multivariate models, Asian, Hispanic/Latine, privately insured, unemployed, those without barriers to social resources, those with chronic conditions, and West Philadelphia residents had lower odds of awareness. Education, income, and anxiety symptoms were not associated with program awareness. Awareness and use of this CHW program is low to moderate among key populations in Philadelphia. Given variations in awareness, scale‐up of CHW programs should include evaluation of program reach, especially to individuals who are not employed and have chronic health conditions.

## Highlights

- Most respondents were unaware of the CHW program—users, however, reported high satisfaction with services.
- Asian and Latine communities show lower CHW program awareness, requiring targeted outreach.
- Unemployed people and those with chronic conditions need better CHW program connection strategies.

## INTRODUCTION

Community Health Workers (CHWs) are frontline public health workers who often come from the same communities they serve, allowing them to share cultural, linguistic, and socioeconomic commonalities with the individuals they support (American Public Health Association, 2014; Olaniran et al., 2017). CHWs have served in a variety of settings across the US for over 70 years. The role originated in the 1950s with grassroots initiatives led by Indigenous workers to support outreach and health education. By the mid‐1960s, the model expanded using “neighborhood health aides,” to address disparities among migrant farmworkers, the urban poor, and Native Americans. These early programs were primarily government‐funded initiatives aimed at promoting community well‐being and alleviating poverty in conjunction with the rise of community health centers. The 1968 establishment of the Indian Health Service Community Health Representative program further formalized the workforce within federal initiatives. (Bovbjerg et al., 2013; Knowles et al., 2023). As unlicensed frontline practitioners, CHWs complete task‐specific training to perform defined roles such as health education, care coordination, cultural competency, motivational interviewing, and navigation of healthcare and social service systems (American Public Health Association, 2014).

CHW programs play a critical role in addressing health disparities by connecting individuals to essential health and social services, particularly in underserved communities (Okasako‐Schmucker et al., 2023). These programs are especially valuable in mitigating the effects of social drivers of health (i.e., economic instability, inadequate housing, food insecurity, and limited access to care) that disproportionately impact racial and ethnic minority populations (Jack et al., 2017; Menendez et al., 2022). Such conditions contribute to chronic stress, unhealthy behaviors, and poor cardiometabolic outcomes (Gooding et al., 2020; M. W. Kreuter, Thompson, et al., 2021; Rosal et al., 2024; Thompson et al., 2019). Unmet social needs also create barriers to timely and preventive healthcare, often resulting in delayed care and worse health outcomes. The recent decline in U.S. life expectancy, especially among younger populations (Harper et al., 2021), underscores the urgency of addressing these structural inequities. Despite their potential, CHW programs remain underutilized, in part due to limited public awareness and uneven access across communities.

CHW support has been linked to significant reductions in emergency department visits (23%–51%) and hospitalizations (21‐50%) among patients with chronic conditions (Jack et al., 2017). By offering culturally competent support, CHWs facilitate connections to medical care, social resources, and health education, ultimately improving health outcomes and reducing emergency healthcare utilization. CHWs serve in diverse settings, including health systems, CBOs, and public health programs (Alhassan & Wills, 2024; Haregu et al., 2024; McCollum et al., 2016). This versatility enables CHWs to adapt to various contexts and contribute to improved health outcomes across communities.

Ensuring that underserved communities have access to CHW services, and are aware of them, is critical to achieving the potential benefits conferred by CHWs. However, awareness and use of CHW programs remain uneven across communities, limiting their potential to improve the lives of underserved populations. For example, in a study of residents of low‐income neighborhoods in El Paso, Texas, Balcazar et al. (Balcazar et al., 2014) found low awareness (20%) and utilization (8%) of a local CHW program focused on promoting cardiovascular health, despite high satisfaction among those who did access them. Research suggests that marginalized populations, including racial/ethnic minorities, experience disproportionate structural barriers to healthcare engagement, including language difficulties, mistrust in healthcare systems, and limited health literacy (Odunsi, 2023; Villagra et al., 2019). Given that CHWs are uniquely positioned to bridge these gaps, understanding the factors that influence CHW awareness is essential for optimizing program reach and effectiveness.

### Study setting

Philadelphia provides a compelling context for examining these issues. As one of the largest cities in the United States with a substantial proportion of racial/ethnic minority residents, Philadelphia faces persistent health disparities, particularly among Black, Hispanic/Latine, and Asian communities. The city has some of the highest rates of chronic diseases, including hypertension, diabetes, and cardiovascular disease, that disproportionately affect these populations (County Health Rankings & Roadmaps, 2024; Philadelphia Department of Public Health, 2023a, 2023b). Furthermore, mental health concerns such as depression and anxiety are widespread (Philadelphia Department of Public Health, 2023b), often exacerbated by socioeconomic stressors like housing instability and food insecurity. Life expectancy varies significantly across neighborhoods, with predominantly Black and low‐income communities experiencing substantially lower life expectancy than more affluent areas (Philadelphia Department of Public Health, 2023a). These disparities highlight the urgent need for targeted community health interventions.

Philadelphia also has a history of community‐driven health initiatives, including CHW programs for adults with multiple chronic conditions recently discharged from the hospital (Edlind et al., 2018; Kangovi et al., 2014) and children with asthma (Bryant‐Stephens et al., 2024). These programs, however, often enroll patients during encounters at health systems, and may have limited reach to individuals with limited healthcare access already. Unlike these programs designed to support high‐acuity populations, the City of Philadelphia's Office of Community Empowerment and Opportunity launched a city‐wide CHW initiative in 2022 aimed at addressing a broader spectrum of health and social needs within community‐based settings. CHWs in this program provide culturally sensitive, trauma‐informed support on health topics such as managing chronic conditions and mental health, as well as assistance with social needs including utility bills, eviction prevention, and referrals to other services. The program operates through partnerships with community‐based organizations, where CHWs provide one‐on‐one assistance and host wellness workshops (City of Philadelphia Office of Community Empowerment and Opportunity, n.d.). Clients are identified through a combination of outreach at community‐based organizations, neighborhood canvassing, and referrals from other social service programs. This city‐wide CHW initiative presents a unique opportunity to evaluate how well these services are reaching minority populations and to identify gaps in awareness and access.

### Study purpose

Although CHWs are intended to bridge gaps in access, it is not fully understood whether awareness of their availability is equitable across diverse populations. Specifically, research examining how awareness of CHW services varies across different sociodemographic groups and by health status remains limited. Without this understanding, targeted outreach efforts to connect those who would benefit most from CHW support may be inefficient or ineffective. Furthermore, while client satisfaction is often reported anecdotally or within specific program evaluations, systematic assessments of overall satisfaction across a broader range of CHW services are still needed to solidify this aspect of their impact. Informed by Andersen's Behavioral Model of Health Services Use (Andersen, 1995), this study seeks to explore these dynamics by assessing the demographic, health, and social factors associated with CHW program awareness among racial/ethnic minority individuals in Philadelphia and to describe patterns of use and satisfaction with services.

## MATERIALS AND METHODS

### Study procedures

Data for this study come from the Philadelphia Community Engagement Alliance (PhillyCEAL) project, a collaborative research initiative to promote health equity across Philadelphia communities. PhillyCEAL is part of the wider National Institutes of Health Community Engagement Alliance (NIH CEAL) network, which includes a diverse coalition of research teams across the United States working to address local health priorities through a community‐partnered approach (Mensah et al., 2024). The network was formed initially as a response to COVID‐19‐related health disparities and has evolved towards a broader focus on partnered approaches to advancing health equity.

For this study, we invited all adult respondents from a previous community survey (Bonett et al., 2024) who identified as racial/ethnic minorities (*n* = 1188) to complete a follow‐up survey in February through April 2024. The original survey, fielded between November 2021 and February 2022, recruited residents of Philadelphia ages 13 and older to complete a web‐based survey focused on experiences and attitudes related to COVID‐19. Full details of the methods used in the original survey have been reported elsewhere (Bonett et al., 2024). The follow‐up survey, on which the present study is based, was a 20‐min web‐based questionnaire that focused on assessing health conditions, healthcare access, engagement with the city's CHW program, and socioeconomic factors affecting Philadelphia residents' wellbeing (Supporting Information S1). The follow‐up survey yielded 550 responses (46.3% response rate). All analyses in the present study are based on data collected in this follow‐up survey, including all demographic variables. Participants were compensated with a US $50 electronic gift card in recognition of their time and effort. All participants reviewed an informed consent document prior to participating in the survey. The Institutional Review Board at the University of Pennsylvania reviewed and approved this study protocol.

### Conceptual framework

Andersen's Behavioral Model of Health Services Use provides a useful framework for understanding the factors influencing access to healthcare services (Andersen, 1995). The model posits that health service utilization is shaped by predisposing factors (e.g., demographic and social characteristics), enabling factors (e.g., socioeconomic resources and geographic accessibility), and need factors (e.g., health conditions and perceived necessity for care). In this study, we adapt the Andersen model to examine how individual and structural barriers are associated with awareness of community‐based health resources such as CHW programs. We extend the model to examine awareness as a fundamental prerequisite for utilization that is similarly influenced by predisposing, enabling, and need factors. As Saurman highlights in her adaptation of the concept of access framework, awareness is a fundamental component of access, as services cannot reach intended populations if those populations do not know the services exist (Saurman, 2016). This adaptation is consistent with studies that have identified lack of awareness as a key barrier to service use (Radhamony et al., 2023).

### Measures

#### Primary and exploratory outcomes

The primary outcome was awareness of the city‐sponsored CHW program operated by the Office of Community Empowerment and Opportunity. Awareness was assessed with a single item asking participants if they were aware of the city's CHW program (yes/no). As exploratory outcomes, we examined program use and satisfaction among those who were aware of the program. Use was assessed by asking whether participants had ever accessed the city‐sponsored CHW program (yes/no). Among those who used services from the city's CHW program, satisfaction was measured on a 5‐point scale from “very dissatisfied” to “very satisfied,” and participants indicated which of the 10 service categories (corresponding to the social needs assessed, described in more detail below) they received assistance with.

#### Demographic characteristics and barriers to health‐related social resources (predisposing and enabling factors)

We collected information on participants' age, gender (categorized as man, woman, or transgender/nonbinary/other), race/ethnicity (categorized as non‐Hispanic Black, Hispanic/Latine, non‐Hispanic Asian, or non‐Hispanic Multiracial/Other), educational attainment (from some high school to graduate degree, collapsed into a binary measure indicating whether a participant had a college degree or not), employment status (employed vs. unemployed, where unemployed included both those seeking employment and those out of the labor force such as students, retirees, individuals with disabilities, and caregivers), and health insurance status (categorized as private, public, or uninsured). Annual household income was assessed using eight categories and collapsed into three levels based on Philadelphia County median household income from the U.S. Census Bureau American Community Survey (Bureau, n.d.): below the local median income (<$50,000), at the local median income ($50,000–$74,999), and above the local median income (≥$75,000). Participants' residential neighborhoods were categorized into six regions of Philadelphia based on zip code: Central, South, Northeast, Northwest, North, and West.

Barriers to health‐related social resources were measured across two main areas: (1) difficulties accessing medical care, social services, mental health support, case management, care coordination, and culturally appropriate health education; and (2) barriers related to navigating the healthcare system, enrolling in health insurance, applying for utility assistance, securing transportation to medical appointments, and obtaining food assistance. Participants reported whether they had experienced any of these 10 barriers to resources in the past 12 months. We created a binary variable (0 = no barriers reported, 1 = one or more barriers reported). Participants were also asked whether they had received assistance from a CHW through the city's program in addressing any of the listed barriers.

### Health status (need factors)

We used a comprehensive checklist of 33 chronic conditions (e.g., asthma, diabetes, hypertension), which were adapted from existing lists of chronic conditions (Erdem et al., 2019; National Library of Medicine Common Data Elements (CDE) Repository, n.d.; PhenX Toolkit, 2025), to assess physical health, and we coded responses into a binary variable indicating the presence (1) or absence (0) of one or more chronic conditions. Mental health was assessed using the Patient Health Questionnaire‐2 (PHQ‐2) for depression screening and the Generalized Anxiety Disorder‐2 scale (GAD‐2) for anxiety screening (Kroenke et al., 2003, 2007). Both measures were dichotomized using the standard clinical cutoff score of ≥3 to create binary variables indicating positive screens for depression and anxiety, respectively (Kroenke et al., 2003, 2007).

### Statistical analysis

#### Data quality assessment

Given the potential for data quality issues in online survey research (Perkel, 2020; Storozuk et al., 2020), we developed a composite score identifying potentially problematic survey responses based on methods used in previous online survey research (Bonett et al., 2024). This score incorporated several indicators: rapid consecutive survey submissions (defined as more than two submissions with start and stop times within 60 s of each other) (Levi et al., 2022; Storozuk et al., 2020), proxy/VPN usage detected through IP address verification (Pozzar et al., 2020; Zhang et al., 2022), mismatches between participants' reported birth nation across survey waves, and discrepancies in participant names and email addresses between survey waves. The name and email comparisons utilized fuzzy string matching to account for minor variations in spelling or formatting. Each indicator contributed one point to the composite data quality score, with higher scores (range: 0‐6) indicating more potential data quality concerns. Since the indicators comprising this score each raise concern about data quality, but do not individually indicate that a response is certainly fraudulent, we only excluded participants with scores of 2 or higher on our data quality score (n = 28 participants excluded). This resulted in a final analytical sample of 522 participants. In all multivariate analyses, we included an indicator for participants who received a data quality score of 1 (i.e., a single data quality issue was found for this participant) as a covariate to adjust for potential systematic biases in survey responses.

#### Analytical methods

We first conducted descriptive analyses of the study sample, including descriptive statistics for demographics, health status, barriers to social resources, and CHW program awareness. We also present descriptions of the frequency of specific barriers addressed by CHWs among program users and reported satisfaction with the program. Given the small amount of missing data, we use complete case analysis for multivariable models.

For our primary analyses examining factors associated with program awareness, we conducted bivariate analyses using chi‐square tests for categorical variables (with Fisher's exact tests when any expected cell frequencies were less than 5) and t‐tests for continuous variables. A multivariate logistic regression model included variables associated with awareness at *p* < .50 in bivariate analyses. Based on guidance for small sample modeling, (Steyerberg et al., 2001) we selected this threshold to balance the need for model parsimony in small samples while preventing the exclusion of potentially important variables. The final model adjusted for race/ethnicity, employment status, insurance type, income, educational attainment, barriers to social resources, neighborhood, chronic condition, anxiety, and the data quality score described above. For categorical variables, global p‐values are calculated using a likelihood ratio test comparing the model with all indicator variables for that predictor to a model without any indicator variables for that predictor. Multicollinearity between predictor variables was assessed using generalized variance inflation factors (GVIF). All analyses were conducted in R version 4.2.0 (R Foundation for Statistical Computing, Vienna, Austria) in May 2025.

## RESULTS

### Sample characteristics

Of the 522 valid respondents, 28 participants (5.4%) had missing data on one or more variables: 26 were missing data on CHW program awareness (our primary outcome), one was missing income level, and one was missing employment status. Characteristics of the sample are presented in Table 1. The mean age of participants was 39.0 years (SD = 11.3, range 18–76). Black participants comprised the majority (*n* = 289, 55.4%), followed by Asian (*n* = 100, 19.2%), Latine (*n* = 100, 19.2%), and Multiracial/Other participants (*n* = 33, 6.3%). The sample was predominantly female (*n* = 366, 70.1%), with men and transgender/nonbinary/other individuals representing 27.8% (*n* = 145) and 2.1% (*n* = 11), respectively. Participants were relatively evenly distributed across income brackets, with 36.1% (*n* = 188) reporting below median income, 28.2% (*n* = 147) at median income, and 35.7% (*n* = 186) above median income. Most participants had either private (*n* = 272, 52.1%) or public insurance (*n* = 165, 31.6%), and 16.3% (*n* = 85) were uninsured. The unemployment rate among participants was 11.9% (*n* = 62). Educational attainment varied across the sample: 7.1% (*n* = 37) had a high school education or less, 16.5% (*n* = 86) had some college but no degree, 56.1% (*n* = 293) had an associate or bachelor's degree, and 20.3% (*n* = 106) had a graduate degree. For analyses, we collapsed education into a binary variable, with approximately three‐quarters of participants (76.4%, *n* = 399) having completed a college degree (associate's degree or higher) and one‐quarter (23.6%, *n* = 123) having less than a college degree. Geographically, participants were distributed across Philadelphia, with the highest representation from North Philadelphia (*n* = 138, 26.4%) and West Philadelphia (*n* = 126, 24.1%).

**Table 1:** Overall sample characteristics and comparison by awareness of CHW program among participants in the PhillyCEAL Year 3 Survey (*n* = 522).

	Overall, *n* = 522	Not aware, *n* = 263	Aware, *n* = 233*	p value
Race/ethnicity (%)				<.001^a^
Asian	100 (19.2)	67 (25.5)	28 (12.0)	
Black	289 (55.4)	115 (43.7)	167 (71.7)	
Latine	100 (19.2)	62 (23.6)	29 (12.4)	
Multiracial/Other	33 (6.3)	19 (7.2)	9 (3.9)	
Gender (%)				.695^a^
Woman	366 (70.1)	184 (70.0)	160 (68.7)	
Man	145 (27.8)	72 (27.4)	69 (29.6)	
Trans/Nonbinary/Other	11 (2.1)	7 (2.7)	4 (1.7)	
Age (mean (SD))	39.01 (11.30)	39.03 (12.69)	39.06 (9.19)	.980^b^
Unemployed (%)	62 (11.9)	50 (19.1)	7 (3.0)	.001^a^
Insurance Status (%)				<.001^a^
Private	272 (52.1)	185 (70.3)	72 (30.9)	
Public	165 (31.6)	70 (26.6)	84 (36.1)	
Uninsured	85 (16.3)	8 (3.0)	77 (33.0)	
Income Level (%)				.275^c^
Above median income	186 (35.7)	97 (36.9)	81 (34.9)	
At median income	147 (28.2)	66 (25.1)	73 (31.5)	
Below median income	188 (36.1)	100 (38.0)	78 (33.6)	
Education, less than college degree (%)	123 (23.6)	69 (26.2)	46 (19.7)	.109^c^
One or more barriers to social resources (%)	380 (72.8)	159 (60.5)	203 (87.1)	<.001^c^
Neighborhood (%)				.001^c^
North	138 (26.4)	60 (22.8)	71 (30.5)	
Central	48 (9.2)	25 (9.5)	22 (9.4)	
Northeast	75 (14.4)	29 (11.0)	41 (17.6)	
Northwest	64 (12.3)	29 (11.0)	32 (13.7)	
South	71 (13.6)	36 (13.7)	30 (12.9)	
West	126 (24.1)	84 (31.9)	37 (15.9)	
One or more chronic conditions (%)	289 (55.4)	172 (65.4)	102 (43.8)	<.001^c^
Depression Screen Positive (%)	95 (18.2)	45 (17.1)	45 (19.3)	.604^c^
Anxiety Screen Positive (%)	112 (21.5)	61 (23.2)	45 (19.3)	.346^c^

*Note*: 26 participants were missing data on CHW awareness.

^a^ Fisher's Exact test.

^b^ Two‐sample t‐test.

^c^ Chi‐squared test.

Regarding health status and barriers to social resources of the sample, nearly three‐quarters (72.8%, *n* = 380) reported experiencing one or more barriers in the past 12 months with a median of 1 social need (interquartile range: 0–2). The most reported social needs were gaining access to medical services and programs (*n* = 104, 19.9%), gaining access to mental health care (*n* = 92, 17.6%), and help navigating the healthcare system (*n* = 83, 15.9%). More than half of the sample (55.4%, *n* = 289) had one or more chronic health conditions, with a median of 1 chronic condition (interquartile range: 0–2). The most reported chronic conditions were asthma (*n* = 93, 17.8%), obesity (*n* = 91, 17.4%), and hypertension (*n* = 84, 16.1%). Mental health screening revealed that 18.2% (*n* = 95) of participants screened positive for depression, while 21.5% (*n* = 112) screened positive for anxiety.

### Program awareness, use, and satisfaction

Of those who responded, 233 participants (47.0%) were aware of the city's CHW program. Among those aware of the program, 71 (30.5%) reported accessing services through the program. Most participants who used the program indicated receiving assistance from a CHW to address at least one of their existing barriers to social resources (*n* = 66, 93.0%). Among those who used the program, satisfaction levels were predominantly positive, with most participants (*n* = 57, 80.3%) being satisfied or very satisfied.

Regarding the specific services participants reported receiving through the CHW program to address barriers to health‐related social resources, access to social services was the most frequently used (*n* = 37, 52.1%), followed by medical services access (*n* = 30, 42.3%), and mental health care access (*n* = 26, 36.6%). Health promotion information and case management support were accessed by 18 (25.4%) and 17 (23.9%) participants, respectively. Health insurance enrollment services were used by 15 participants (21.1%), while healthcare system navigation was accessed by 14 participants (19.7%). Less frequently used services included food/grocery assistance (*n* = 12, 16.9%), utility assistance (*n* = 10, 14.1%), and transportation referrals (*n* = 7, 9.9%).

### Factors associated with CHW program awareness

Results of the multivariate logistic regression analysis are presented in Table 2. In multivariate logistic regression analysis, race/ethnicity was significantly associated with program awareness, with Asian (aOR = 0.44, 95% CI: 0.22–0.84, *p* = .014) and Hispanic/Latine participants (aOR = 0.47, 95% CI: 0.25–0.88, *p* = .019) having lower odds of program awareness compared to Black participants. Insurance status was associated with awareness, with publicly insured (aOR = 4.89, 95% CI: 2.80–8.70, *p* < .001) and uninsured participants (aOR = 17.81, 95% CI: 7.62–46.88, *p* < .001) showing significantly higher odds of program awareness compared to those with private insurance. Employment status was significantly associated with program awareness, with unemployed participants having lower odds of awareness compared to employed participants (aOR = 0.10, 95% CI: 0.04–0.25, *p* < .001). Participants reporting one or more barriers to health‐related social resources had significantly higher odds of program awareness (aOR = 2.40, 95% CI: 1.37–4.27, *p* = .002), while those with one or more chronic conditions had lower odds of awareness (aOR = 0.49, 95% CI: 0.30–0.81, *p* = .006). Geographic location also played a role, with residents of West Philadelphia showing significantly lower odds of program awareness compared to North Philadelphia residents (aOR = 0.27, 95% CI: 0.14–0.52, *p* < .001). Education level, income level, and anxiety symptoms were not significantly associated with program awareness in the multivariate model. Assessment of multicollinearity revealed no concerning relationships between predictor variables (GVIF <1.5 for all predictors).

**Table 2:** Logistic regression modeling awareness of CHW program among participants in the PhillyCEAL Year 3 survey (*n* = 494).

	aOR*	95% CI, LL	95% CI, UL	p‐value
Race/ethnicity	‐‐‐	‐‐‐	‐‐‐	.025^a^
Black	REF	REF	REF	REF
Asian	0.44	0.22	0.84	.014
Latine	0.47	0.25	0.88	.019
Multiracial/Other	0.67	0.25	1.66	.398
Unemployed	0.10	0.04	0.25	<.001
Insurance Status	‐‐‐	‐‐‐	‐‐‐	<.001^a^
Private	REF	REF	REF	REF
Public	4.89	2.80	8.70	<.001
Uninsured	17.81	7.62	46.88	<.001
Income Level	‐‐‐	‐‐‐	‐‐‐	.533^a^
Above median income	REF	REF	REF	REF
At median income	0.96	0.53	1.72	.896
Below median income	0.72	0.39	1.33	.299
Education, less than college degree	0.58	0.32	1.03	.067
One or more barriers to social resources	2.40	1.37	4.27	.002
Neighborhood	‐‐‐	‐‐‐	‐‐‐	.002^a^
North	REF	REF	REF	REF
Central	0.55	0.23	1.32	.182
Northeast	1.00	0.46	2.16	.999
Northwest	0.70	0.32	1.51	.368
South	0.52	0.23	1.14	.105
West	0.27	0.14	0.52	<.001
One or more chronic conditions	0.49	0.30	0.81	.006
Anxiety Screen Positive	0.94	0.54	1.63	.819

*variables significant a *p* < .50 in bivariate tests were included in multivariate model. Model included adjustment for data quality score.

^a^ global p‐value, calculated using likelihood ratio chi‐squared test.

## DISCUSSION

Awareness plays a crucial role in the utilization of public health services, influencing individuals' decisions to seek and utilize available benefits. Our study examined the awareness and use of the City of Philadelphia's CHW services—a critical public health resource, particularly for communities with a high proportion of unmet social needs. While CHWs offer established benefits in connecting community members to essential services, our findings reveal a significant opportunity to enhance both awareness and use of these services. As highlighted by Saurman in her adaptation of the concept of access framework, awareness is a fundamental component of access that is required for health and social services to effectively reach the intended populations (Saurman, 2016). We found that over half of the participants in our sample were unaware of the city‐wide CHW program. This lack of awareness presents a substantial barrier to service utilization and highlights a critical area for intervention. Even though people often become aware of other CHW programs during healthcare encounters or in other settings, early awareness about public health services such as the city‐wide CHW program remains critical—empowering people to seek support before health issues escalate and helping to mitigate the social stressors that can negatively influence health outcomes.

Our study revealed variations in CHW program awareness across several key demographic groups. Aligned with the Andersen Behavioral Model of Health Services Use (Andersen, 1995), these findings can be categorized by predisposing, enabling, and need factors. Predisposing factors, such as race and ethnicity, were associated with awareness disparities, as Hispanic/Latine and Asian participants were less aware of CHW services compared to Black residents. Although the program employed CHWs from both Asian and Hispanic/Latine communities, their reach was likely limited due to the wide geographic area served by the program and the multiple ethnic enclaves that exist across Philadelphia. Philadelphia has substantial immigrant populations within these communities, with approximately 33% of the city's foreign‐born residents identifying as Asian and 27% identifying as Latine (The Pew Charitable Trusts, 2024a). Previous research has found that communities with high numbers of immigrants and refugees face heightened barriers to accessing social services, even when language services are available (Byrow et al., 2020; Clough et al., 2013). Addressing these gaps requires culturally responsive outreach efforts, including partnerships with existing organizations and community leaders in Asian and Latine communities (Baezconde‐Garbanati et al., 2023; Pringle et al., 2022), which could help to increase awareness of CHW programs and link residents to health resources.

Enabling factors, such as geographic location, employment status, and barriers to health‐related social resources, were also associated with CHW program awareness. West Philadelphia residents were significantly less aware of the City CHW program compared to those in North Philadelphia. The reasons for this geographic disparity are unclear from our data and warrant further investigation, particularly given that both neighborhoods face similar challenges including poverty and disinvestment. Future research should examine whether CHW program implementation (e.g., CBO partnerships, outreach strategies, staffing) varied across neighborhoods in ways that affected awareness.

Additionally, unemployed individuals had lower odds of being aware of CHW services, indicating a missed opportunity to connect this group with critical resources that could address their social needs. Given that Philadelphia has high levels of poverty and unemployment (The Pew Charitable Trusts, 2024b), integrating CHW outreach into a larger number of workforce development programs, food assistance programs, and neighborhood health centers could improve awareness among populations that stand to benefit the most from their services. Participants reporting one or more barriers to social resources had significantly higher odds of CHW program awareness suggesting that those experiencing challenges with healthcare access, transportation, or food access may be more likely to seek out or be connected with CHW services. Unmet social needs rarely exist in isolation; they tend to cluster, creating a complex web of challenges (e.g., unemployment leading to financial strain, which then contributes to food insecurity) (M. Kreuter et al., 2020). This interconnectedness increases the risk of adopting adverse health behaviors (Thompson et al., 2019) and experiencing poorer health outcomes in the long run. Therefore, the significant associations we found highlight critical opportunities for targeted CHW outreach and intervention.

Need factors were also found to be associated with CHW program awareness. Participants with one or more chronic conditions had significantly lower odds of awareness, which is concerning given that individuals with chronic conditions could particularly benefit from CHW support. The relatively young average age of participants (late 30s) in our sample may partially explain these findings, as younger adults with chronic conditions might not yet perceive a need for additional services from CHWs and may be less likely to seek out these supportive services. However, research has shown that even a single chronic condition in young adults can significantly impact long‐term health trajectories (Ge et al., 2019).

Our findings align with existing research demonstrating high levels of client satisfaction with CHW services (Carson et al., 2022; Chang et al., 2021). Client satisfaction plays a crucial role in health and social service delivery, influencing follow‐up and retention in care, and is essential for achieving optimal clinical outcomes (Mundorf et al., 2017). Positive experiences can foster trust and rapport which are crucial for long‐term engagement, especially in populations facing health disparities. Although patient satisfaction is a multifaceted outcome, future studies using longitudinal methods may reveal whether patient satisfaction with CHW services is associated with sustained engagement with CHW services and less unmet social needs over time.

This study has several limitations. First, our sample was relatively young, predominantly female, and recruited through convenience sampling, which may limit the generalizability of our findings. Therefore, our findings regarding CHW awareness rates should be interpreted as estimates within our sampled population rather than robust estimates of citywide prevalence. However, this highlights an opportunity to increase awareness about CHW services within this demographic. Second, despite rigorous data quality checks, online survey methodologies are subject to limitations such as self‐report bias and fraudulent responses. Third, the cross‐sectional nature of our study limits our ability to determine the directionality of the observed associations. For example, it is unclear whether individuals experiencing barriers are more likely to become aware of CHW services, or whether awareness of CHW programs enables individuals to better articulate and report their unmet needs. Longitudinal research is needed to disentangle this relationship. Future research should employ mixed‐methods approaches, including qualitative interviews and focus groups, to further explore barriers to CHW awareness and identify tailored strategies for increasing engagement. Additionally, incorporating geographic analysis to examine neighborhood‐level disparities in CHW access would enhance understanding of location‐based inequities.

Our study underscores Philadelphia's ongoing racial, socioeconomic, and geographic disparities in healthcare access and reveals differential awareness of a public health service intended to mitigate these inequities. Despite these variations, the high level of satisfaction among those who access CHW services underscores their potential impact on addressing social needs in our city. These findings provide a roadmap for targeted outreach strategies to improve program awareness and use, particularly among underserved populations. Expanding culturally tailored outreach, integrating CHW programs into employment and social service settings, and leveraging community partnerships will be critical next steps in ensuring equitable access to these vital public health resources.

## AUTHOR CONTRIBUTIONS

Conceptualization: SB, CA, JB, AC; Data curation: SB, HY; Formal Analysis: SB, CA, JB; Funding acquisition: JB, AM; Investigation: SB, CA, JB, AC, AST, THL, KG, AM; Methodology: SB; Supervision: JB, AM; Writing – original draft: SB, CA; Writing – review & editing: SB, CA, JB, AC, HY, AST, THL, KG, AM.

## CONFLICT OF INTEREST STATEMENT

The authors declare no conflicts of interest.

## ETHICAL APPROVAL

The Institutional Review Board at the University of Pennsylvania reviewed and approved this study protocol.

## Supporting information

Supporting File 1.

## Data Availability

The data that support the findings of this study are available from the corresponding author upon reasonable request.
