# Supplementary material for: Awareness and use of a community health worker program among racial/ethnic minority adults in Philadelphia: Results from a cross‐sectional community health survey
Source: Am J Community Psychol. 2026 Jul 28;78(1-2):234–44. doi: 10.1002/ajcp.70092 (PMC13614425; doi:10.1002/ajcp.70092)
Supplement: Supplementary file 1 [file AJCP-78-234-s001.docx]

**This survey should take approximately 30 minutes to complete.**  Your participation is completely voluntary, and your responses will be kept confidential.

Please indicate the state and county you live in.  For Puerto Rico, indicate that as the state.

State

County/Municipality

▼ Alabama ... Wyoming ~ Weston County

Please indicate the zip code where you live.

________________________________________________________________

What month were you born?

▼ January ... December

What year were you born?

▼ 1923 ... 2023

Age:

________________________________________________________________

Where were you born?

- In the United States
- In a U.S. Territory
- Outside the United States, please specify country: __________________________________________________

Which state were you born in?

▼ Alabama ... Wyoming

Which Territory were you born in?

▼ Puerto Rico ... Northern Mariana Islands

What was your sex assigned at birth on your birth certificate?

- Female
- Male

What is your current gender?

- Man
- Woman
- Non-binary
- Transgender
- None of these describe me, and I’d like to consider additional options

Are any of these a closer description to your gender identity?

- Trans Man/Transgender Man/FTM
- Trans Woman/Transgender Woman/MTF
- Genderqueer
- Genderfluid
- Gender variant
- Questioning or unsure of your gender identity
- None of these describe me, and I want to specify __________________________________________________

Are you:

- Married
- Divorced
- Widowed
- Separated
- Never married
- A member of an unmarried couple

Which of the following best represents how you think of yourself?

- Straight, that is, not gay or lesbian, etc.
- Gay
- Lesbian
- Bisexual
- None of these describe me, and I’d like to see additional options

Are any of these a closer description of how you think of yourself?

- Queer
- Polysexual, omnisexual, sapiosexual, or pansexual
- Asexual
- Two-spirit
- Have not figured out or are in the process of figuring out your sexuality
- Mostly straight, but sometimes attracted to people of your own sex
- Do not think of yourself as having sexuality
- Do not use labels to identity yourself
- Don’t know the answer
- No, I mean something else: __________________________________________________

Are you Hispanic/Latino/a or Spanish origin?

- ⊗No, not of Hispanic, Latino, or Spanish origin
- Yes, Brazilian
- Yes, Cuban
- Yes, Colombian
- Yes, Dominican
- Yes, Guatemalan
- Yes, Honduran
- Yes, Mexican, Mexican American, Chicano
- Yes, Puerto Rican
- Yes, Salvadoran
- Other, please specify (for example, Ecuadorian, Nicaraguan, Peruvian, Spaniard, Venezuelan): __________________________________________________

Which of the following best describes your race? (Select all that apply.)

- American Indian or Alaska Native
- Asian
- Black, African American, or African
- Native Hawaiian/Pacific Islander
- White

American Indian/Alaskan Native - Which best applies to you?

- Enrolled in a federally recognized tribe (Please Specify:) __________________________________________________
- Enrolled in a state recognized tribe (Please Specify:) __________________________________________________
- Eligible for enrollment, but I am not enrolled in my tribe (Please Specify:) __________________________________________________
- Not enrolled, but I am a descendent of an American Indian or Alaska Native tribe
- ⊗Not applicable, my Indigenous People/Community do not have tribal enrollment
- Other, please specify: __________________________________________________

Is your location:

- Urban
- Rural
- On a Reservation
- Other, please specify: __________________________________________________

Asian - Which best applies to you?

- Asian Indian
- Bangladeshi
- Burmese
- Bhutanese
- Cambodian
- Chinese, except Taiwanese
- Filipino
- Hmong
- Indonesian
- Japanese
- Korean
- Laotian
- Malaysian
- Mongolian
- Nepalese
- Okinawan
- Pakistani
- Sri Lankan
- Taiwanese
- Thai
- Vietnamese
- Other, please specify: __________________________________________________

Black/African American/African - Which best applies to you?

- African American
- Barbadian
- Ethiopian
- Ghanaian
- Haitian
- Jamaican
- Liberian
- Nigerian
- Somali
- South African
- Trinidadian and Tobagonian
- Other, please specify: __________________________________________________

Native Hawaiian/Pacific Islander - Which best applies to you?

- Chamorro
- Chuukese
- Fijian
- Marshallese
- Native Hawaiian
- Palauan/Belauan
- Pohnpeian
- Samoan
- Tongan
- Maori
- Kosraen
- Yapese
- Other, please specify: __________________________________________________

White - Which best applies to you?

- English
- French
- German
- Irish
- Italian
- Polish
- Other, please specify: __________________________________________________

What is the highest degree or level of school you have completed?

- Less than high school (for example, middle or elementary school)
- Some high school
- High school graduate
- GED
- Some college – no degree
- Associate’s or technical degree (for example, AA or AS)
- Bachelor’s degree (for example, BA, BS, or AB)
- Graduate degree (for example, MA or PhD)
- None

How many people live in your household right now? Include yourself, any other adults, and any children.

|  | Number |
| --- | --- |
| Adults (age 18 or older) |  |
| Minors (younger than age 18) |  |

Do you speak a language other than English at home?

- Yes
- No

Which language(s)?

________________________________________________________________

How well do you speak English?

- Like a Native
- Very well
- Well
- Not well
- Not at all
- Don't Know

What was your total household income before taxes?

- Less than $15,000
- $15,000 - $19,999
- $20,000 - $24,999
- $25,000 - $34,999
- $35,000 - $49,999
- $50,000 - $74,999
- $75,000 - $99,999
- $100,000 and above

***The following section will ask questions related to your social well-being.***

What kind of place do you go most often for medical care?

- A doctor’s office (this could be in a health system or HMO)
- A VA Medical Center or VA outpatient clinic
- Emergency Room
- Some other place
- Telehealth or Telemedicine
- Walk-in Clinic or urgent care center or retail clinic in a pharmacy or grocery store
- There is no one place I go to most often

What kind of health insurance or health plan do you have now? Select all that apply.

- ⊗I don’t have health insurance or health plan
- Indian Health Service
- Medicaid, Medical Assistance (MA), CHIP or kid’s state insurance
- Medicare
- Military health care (e.g., Tricare)
- Private health insurance (through a job, school or bought directly)
- Other, please specify: __________________________________________________

During the past 12 months, how often have you waited to get medical care, because of cost?

- Always
- Usually
- Sometimes
- Rarely
- Never
- Not applicable – I didn’t need medical care in the past 12 months.

How often do you need someone to help you read written information from your doctor or drug store?

- Always
- Usually
- Sometimes
- Rarely
- Never

If for any reason you need help with day-to-day activities such as bathing, preparing meals, shopping, managing finances, etc., do you get the help you need?

- I don’t need any help
- I get all the help I need
- I could use a little more help
- I need a lot more help

What type of housing do you currently live in?

- Apartment
- Single family house
- Homeless
- Shelter
- Other: __________________________________________________

Do you own or rent your home?

- Own
- Rent
- Other arrangement: __________________________________________________

In the last 12 months, how often have you been worried about not being able to pay your rent or mortgage?

- Always
- Usually
- Sometimes
- Rarely
- Never

In the last 12 months, how often did you cut the size of your meals or skip meals because there wasn't enough money for food?

- Always
- Usually
- Sometimes
- Rarely
- Never

Do you have money left over at the end of the month?

- Always
- Often
- Sometimes
- Rarely
- Never

Which best describes your current employment status (select all that apply)?

- Working for pay—part time (less than 40 hours a week)
- Working for pay—full time (40 hours a week or more)
- Unemployed and looking for a job
- Unemployed and NOT looking for a job
- Other, please specify: __________________________________________________

Was there ever a time when you felt you would have gotten better medical care if you had belonged to a different race or ethnic group?

- Yes
- No

How easy would it be for you to find a job with another employer with approximately the same income and fringe benefits you now have? Would you say very easy, somewhat easy, or not easy at all?

- Very easy
- Somewhat easy
- Not easy at all
- Don’t know
- Does not apply

Please indicate whether you have ever needed any of the following services:

- Gaining access to medical services and programs
- Gaining access to social services or programs
- Gaining access to mental health care
- Securing case management and care coordination support
- Help navigating the healthcare system
- Accessing culturally appropriate health promotion information or education (for example, workshops, classes, online guides, or other resources or activities related to improving your health)
- Help getting enrolled into a health insurance program
- Support with utility assistance program applications
- Referrals to transportation services for medical appointments
- Receiving food or groceries for you or your family
- Other; please specify __________________________________________________

Please indicate whether you have needed any of the following services in the past 12 months:

- Gaining access to medical services and programs
- Gaining access to social services or programs
- Gaining access to mental health care
- Securing case management and care coordination support
- Help navigating the healthcare system
- Accessing culturally appropriate health promotion information or education (for example, workshops, classes, online guides, or other resources or activities related to improving your health)
- Help getting enrolled into a health insurance program
- Support with utility assistance program applications
- Referrals to transportation services for medical appointments
- Receiving food or groceries for you or your family
- Other; please specify __________________________________________________

Are you aware that the City of Philadelphia has a Community Health Worker program?

- Yes
- No
- Don't Know

Have you ever accessed the City of Philadelphia Community Health Worker program?

- Yes
- No
- Don't Know

How satisfied were you with the help you received from the Community Health Worker?

- Very Satisfied
- Satisfied
- Neither satisfied nor dissatisfied
- Dissatisfied
- Very dissatisfied

Which of the following services did your Community Health Worker help with?

- Gaining access to medical services and programs
- Gaining access to social services or programs
- Gaining access to mental health care
- Securing case management and care coordination support
- Help navigating the healthcare system
- Accessing culturally appropriate health promotion information or education (for example, workshops, classes, online guides, or other resources or activities related to improving your health)
- Help getting enrolled into a health insurance program
- Support with utility assistance program applications
- Referrals to transportation services for medical appointments
- Receiving food or groceries for you or your family
- Other; please specify __________________________________________________

How much do you trust each of these sources to provide correct health information?

|  | Not at all | A little | A great deal | Does not apply |
| --- | --- | --- | --- | --- |
| Your doctor or healthcare provider |  |  |  |  |
| Your faith leader (e.g., priest, minister, rabbi) |  |  |  |  |
| Family and Friends |  |  |  |  |
| People you go to work or class with or other people you interact with regularly |  |  |  |  |
| News on the radio |  |  |  |  |
| News on TV |  |  |  |  |
| News in the newspaper |  |  |  |  |
| News online |  |  |  |  |
| Social media |  |  |  |  |
| Federal government health agencies such as the Centers for Disease Control and Prevention (CDC), the National Institutes of Health (NIH) and the Food and Drug Administration (FDA) |  |  |  |  |
| State and/or local government |  |  |  |  |
| Tribal leadership |  |  |  |  |
| Community health workers (e.g., peer educators, promotores de salud, community ambassadors or coaches) |  |  |  |  |
| A community organization that provides services and assistance where you live |  |  |  |  |

Over the **last 14 days**, how often have you been bothered by having little interest or pleasure in doing things? Would you say…

- Not at all
- Several days
- More than half the days
- Nearly every day

Over the **last 14 days**, how often have you been bothered by feeling down, depressed, or hopeless? Would you say…

- Not at all
- Several days
- More than half the days
- Nearly every day

Over the **last 14 days**, how often have you been bothered by the following problems: Feeling nervous, anxious, or on edge? Would you say…

- Not at all
- Several days
- More than half the days
- Nearly every day

Over the **last 14 days**, how often have you been bothered by the following problems: Not being able to stop or control worrying? Would you say…

- Not at all
- Several days
- More than half the days
- Nearly every day

| Page Break |  |
| --- | --- |

***The next questions are about how you feel about different aspects of your life. For each one, tell me how often you feel that way.***

First, how often do you feel that you lack companionship?

- Never
- Some of the time
- Often
- All of the time

How often do you feel left out?

- Never
- Some of the time
- Often
- All of the time

How often do you feel isolated from others?

- Hardly Ever
- Some of the time
- Often

Please check all of the medical conditions that a doctor, nurse, or other healthcare professional has told you that you have.

- Alzheimer’s Disease
- Asthma
- Autoimmune/Immunocompromised Condition
- Cancer, please specify type: __________________________________________________
- Chronic Kidney Disease
- Chronic Musculoskeletal Conditions (e.g., Back Pain, Osteoarthritis, and Osteoporosis)
- Coronary Disease
- Dementia
- Diabetes (Type I)
- Diabetes (Type II)
- Epilepsy
- Heart Failure
- Hepatitis
- High Blood Pressure or Hypertension
- High Cholesterol
- Immune Deficiency/HIV/AIDS
- Long COVID
- Multiple Sclerosis
- Obesity
- Other Chronic Liver Disease
- Other Chronic Neurological Condition/Nervous System (e.g. Parkinson’s Disease, Migraine, Head Injury)
- Other Chronic Lung or Respiratory Disease or Disorders (e.g., COPD, Emphysema)
- Other Heart Disease, Cardiovascular Disease, or Vascular Disease
- Depression
- Anxiety
- Other Psychological and/or Psychiatric Condition (e.g., Bipolar, Dissociation, Eating)
- Rheumatologic Conditions (e.g., Rheumatoid Arthritis, Systemic Lupus Erythematosus, Vasculitis)
- Sickle Cell Disease
- Sleep Disorders (e.g., Insomnia, Sleep Apnea, Narcolepsy, Restless Legs Syndrome)
- Solid Organ Transplant Recipient
- Substance Use Disorders (e.g., Alcohol, Drugs, Smoking)
- Stroke
- Thrombotic Disorders
- Other Medical Conditions (please specify) __________________________________________________
- ⊗None of the above

Do you currently receive any treatments or interventions (medications, classes/educational sessions, or coaching/counseling) for the condition? Select all that apply.

|  | Yes, medications | Yes, classes/educational sessions | Yes, coaching/counseling |
| --- | --- | --- | --- |
| Alzheimer’s Disease |  |  |  |
| Asthma |  |  |  |
| Autoimmune/Immunocompromised Condition |  |  |  |
| Cancer, please specify type: |  |  |  |
| Chronic Kidney Disease |  |  |  |
| Chronic Musculoskeletal Conditions (e.g., Back Pain, Osteoarthritis, and Osteoporosis) |  |  |  |
| Coronary Disease |  |  |  |
| Dementia |  |  |  |
| Diabetes (Type I) |  |  |  |
| Diabetes (Type II) |  |  |  |
| Epilepsy |  |  |  |
| Heart Failure |  |  |  |
| Hepatitis |  |  |  |
| High Blood Pressure or Hypertension |  |  |  |
| High Cholesterol |  |  |  |
| Immune Deficiency/HIV/AIDS |  |  |  |
| Long COVID |  |  |  |
| Multiple Sclerosis |  |  |  |
| Obesity |  |  |  |
| Other Chronic Liver Disease |  |  |  |
| Other Chronic Neurological Condition/Nervous System (e.g. Parkinson’s Disease, Migraine, Head Injury) |  |  |  |
| Other Chronic Lung or Respiratory Disease or Disorders (e.g., COPD, Emphysema) |  |  |  |
| Other Heart Disease, Cardiovascular Disease, or Vascular Disease |  |  |  |
| Depression |  |  |  |
| Anxiety |  |  |  |
| Other Psychological and/or Psychiatric Condition (e.g., Bipolar, Dissociation, Eating) |  |  |  |
| Rheumatologic Conditions (e.g., Rheumatoid Arthritis, Systemic Lupus Erythematosus, Vasculitis) |  |  |  |
| Sickle Cell Disease |  |  |  |
| Sleep Disorders (e.g., Insomnia, Sleep Apnea, Narcolepsy, Restless Legs Syndrome) |  |  |  |
| Solid Organ Transplant Recipient |  |  |  |
| Substance Use Disorders (e.g., Alcohol, Drugs, Smoking) |  |  |  |
| Stroke |  |  |  |
| Thrombotic Disorders |  |  |  |
| Other Medical Conditions (please specify) |  |  |  |
| ⊗None of the above |  |  |  |

Please indicate how often the medical condition limits your activities.

|  | Never | Rarely | Some of the time | Often | All of the time |
| --- | --- | --- | --- | --- | --- |
| Alzheimer’s Disease |  |  |  |  |  |
| Asthma |  |  |  |  |  |
| Autoimmune/Immunocompromised Condition |  |  |  |  |  |
| Cancer, please specify type: |  |  |  |  |  |
| Chronic Kidney Disease |  |  |  |  |  |
| Chronic Musculoskeletal Conditions (e.g., Back Pain, Osteoarthritis, and Osteoporosis) |  |  |  |  |  |
| Coronary Disease |  |  |  |  |  |
| Dementia |  |  |  |  |  |
| Diabetes (Type I) |  |  |  |  |  |
| Diabetes (Type II) |  |  |  |  |  |
| Epilepsy |  |  |  |  |  |
| Heart Failure |  |  |  |  |  |
| Hepatitis |  |  |  |  |  |
| High Blood Pressure or Hypertension |  |  |  |  |  |
| High Cholesterol |  |  |  |  |  |
| Immune Deficiency/HIV/AIDS |  |  |  |  |  |
| Long COVID |  |  |  |  |  |
| Multiple Sclerosis |  |  |  |  |  |
| Obesity |  |  |  |  |  |
| Other Chronic Liver Disease |  |  |  |  |  |
| Other Chronic Neurological Condition/Nervous System (e.g. Parkinson’s Disease, Migraine, Head Injury) |  |  |  |  |  |
| Other Chronic Lung or Respiratory Disease or Disorders (e.g., COPD, Emphysema) |  |  |  |  |  |
| Other Heart Disease, Cardiovascular Disease, or Vascular Disease |  |  |  |  |  |
| Depression |  |  |  |  |  |
| Anxiety |  |  |  |  |  |
| Other Psychological and/or Psychiatric Condition (e.g., Bipolar, Dissociation, Eating) |  |  |  |  |  |
| Rheumatologic Conditions (e.g., Rheumatoid Arthritis, Systemic Lupus Erythematosus, Vasculitis) |  |  |  |  |  |
| Sickle Cell Disease |  |  |  |  |  |
| Sleep Disorders (e.g., Insomnia, Sleep Apnea, Narcolepsy, Restless Legs Syndrome) |  |  |  |  |  |
| Solid Organ Transplant Recipient |  |  |  |  |  |
| Substance Use Disorders (e.g., Alcohol, Drugs, Smoking) |  |  |  |  |  |
| Stroke |  |  |  |  |  |
| Thrombotic Disorders |  |  |  |  |  |
| Other Medical Conditions (please specify) |  |  |  |  |  |
| ⊗None of the above |  |  |  |  |  |

Do you now smoke cigarettes?

- Every day
- Some days
- Not at all

On average, on the days you smoke, how many cigarettes do you smoke each day?

________________________________________________________________

Do you now use e-cigarettes (vaping)?

- Every day
- Some days
- Not at all

On average, how many days per week do you engage in moderate to strenuous exercise (like a brisk walk)?

- 0 days
- 1 day
- 2 days
- 3 days
- 4 days
- 5 days
- 6 days
- 7 days

On average, how many minutes do you engage in exercise at this level per week? Please specify.

________________________________________________________________

***These questions are about the different kinds of foods you ate or drank during the PAST MONTH, that is, the past 30 days. When answering, please include meals and snacks eaten at home, at work or school, in restaurants, and anyplace else.***

During the past month, how often did you eat FRUIT? Count fresh, frozen, or canned fruit. Do not count juices.

- Never
- 1-2 times per week
- 3-4 times per week
- 5-6 times per week
- More than 6 times per week
- Don’t know

During the past month, how often did you eat a green leafy or lettuce SALAD, with or without other vegetables? Count Spinach, Kale, Lettuce, Collard Green.

- Never
- 1-2 times per week
- 3-4 times per week
- 5-6 times per week
- More than 6 times per week
- Don’t know

During the past month, how often did you eat OTHER VEGETABLES? Examples of other vegetables include tomatoes, string beans, carrots, corn, sweet potatoes, cabbage, bean sprouts, and broccoli.

- Never
- 1-2 times per week
- 3-4 times per week
- 5-6 times per week
- More than 6 times per week
- Don’t know

During the past month, how often did you drink sweetened fruit drinks, sports or energy drinks, such as Kool-aid, lemonade, Hi-C, cranberry drink, Gatorade, Red Bull or Vitamin Water? Sweetened Tea? Include fruit juices you made at home and added sugar to.

- Never
- 1-2 times per week
- 3-4 times per week
- 5-6 times per week
- More than 6 times per week
- Don’t know

During the past month, how often did you drink regular soda or pop that contains sugar? Do not include diet soda.

- Never
- 1-2 times per week
- 3-4 times per week
- 5-6 times per week
- More than six time per week
- Don’t know

What is your best estimate of the number of days you drank alcohol during the past 30 days? Please specify

________________________________________________________________

On the days that you drank during the past 30 days, how many drinks did you usually have each day? Count as a drink a can or bottle of beer; a wine cooler or a glass of wine, champagne, or sherry; a shot of liquor or a mixed drink or cocktail.

________________________________________________________________

This concludes our survey, thank you for participating!
